# Mental Health Training for Community Health Workers in Cancer Care: A Narrative Review

**DOI:** 10.3390/healthcare13192500

**Published:** 2025-10-01

**Authors:** Mercedes Ramirez-Ruiz, Jovanny Díaz-Rodríguez, Normarie Torres-Blasco, Nelmit Tollinchi-Natali, Dayaneira Rivera-Alers, Jean Robles-Gutiérrez, Jessenia Mercado-Negrón, Gabriela Marrero-Quiñones, Paola del Río-Rodríguez, Guillermo Laporte-Estela, Zindie Rodríguez-Castro, Cynthia Cortes-Castro, Cristina I. Pena-Vargas, Rosario Costas-Muñiz, Paula Cupertino, Julio Jiménez-Chávez, Eliut Rivera-Segarra, Guillermo N. Armaiz-Pena, Eida M. Castro-Figueroa

**Affiliations:** 1 Ponce Research Institute, Ponce, PR 00716, USA; mramirez@psm.edu (M.R.-R.); jovadiaz@psm.edu (J.D.-R.); normarietorres@psm.edu (N.T.-B.); ntollinchi@psm.edu (N.T.-N.); dayrivera@psm.edu (D.R.-A.); gmarrero22@stu.psm.edu (G.M.-Q.); zrodriguez@psm.edu (Z.R.-C.); ccortes@psm.edu (C.C.-C.); cpena@psm.edu (C.I.P.-V.); jcjimenez@psm.edu (J.J.-C.); elrivera@psm.edu (E.R.-S.); garmaiz@psm.edu (G.N.A.-P.); 2 Public Health Program, Ponce Health Sciences University, Ponce, PR 00716, USA; 3 School of Behavioral and Brain Sciences, Ponce Health Sciences University, Ponce, PR 00716, USA; jrobles23@stu.psm.edu (J.R.-G.); jmercado23@stu.psm.edu (J.M.-N.); pdelrio22@stu.psm.edu (P.d.R.-R.); glaporte22@stu.psm.edu (G.L.-E.); 4 School of Medicine, Ponce Health Sciences University, Ponce, PR 00716, USA; 5 Department of Psychiatry and Behavioral Sciences, Memorial Sloan Kettering Cancer Center, New York, NY 10065, USA; costasmr@mskcc.org; 6 Department of Psychiatry Weill Cornel Medical College, Weill Cornell Medicine, New York, NY 10065, USA; 7 Wilmot Cancer Institute, University of Rochester Medical Center, Rochester, NY 14642, USA; paula_cupertino@urmc.rochester.edu; 8 School of Dental Medicine, Ponce Health Sciences University, Ponce, PR 00716, USA

**Keywords:** mental health cancer, community mental health workers, training, psychological well-being, narrative review, health promotion

## Abstract

Background/Objectives: Lay Community Health Workers (CHWs) play a critical role in reducing mental health disparities, particularly among underserved and vulnerable populations, by bridging gaps in care and promoting mental well-being. This narrative review aimed to identify and characterize training programs designed for CHWs, with a focus on those targeting cancer patients and individuals with chronic conditions. Methods: A comprehensive literature search was conducted across databases including PubMed, EBSCOhost, Scielo, Redalyc, and Google Scholar. From an initial pool of 10,372 references, 27 relevant articles were selected, encompassing research articles, training materials, curricula, and other resources. Results: The identified training methods included role-playing, instructional videos, and manuals designed to equip CHWs with skills in mental health intervention, prevention, management, education, and stigma reduction. Training programs also incorporated evidence-based interventions and psychological skill training. This review highlights a notable gap in research on CHW-led interventions in cancer palliative care and mental health. Conclusions: The findings support the development of a specialized mental health training program tailored for CHWs working with cancer survivors, to enhance their capacity to address mental health challenges, reduce stigma, and promote psychological well-being. Future efforts involve developing a training intervention for CHWs to support the needs of cancer survivors.

## 1. Introduction

### 1.1. Background on Community Health Workers

Mental health disorders represent a critical global challenge due to their high prevalence, substantial impact on quality of life, and significant socio-economic burden [1]. Approximately 25% of individuals are estimated to experience at least one mental health or behavioral condition during their lifetime [2]. Depression, identified by the World Health Organization as a leading cause of global disease burden, disproportionately affects low- and middle-income countries [3]. The coexistence of multiple chronic conditions, including both physical and mental disorders, further complicates patient care and management [4].

Although there are effective treatments available, many people with mental health conditions still do not receive the care they need [5]. This gap in care is especially pronounced in underserved groups such as Hispanic communities, where access to quality mental health services is hindered by factors like stigma, poverty, and low levels of health literacy [6,7].

A critical factor contributing to the mental health treatment gap is the shortage of trained mental health providers [8]. To address this challenge, delegating primary prevention efforts to CHWs has been proposed as a strategy to expand service coverage and improve access to culturally competent care [9]. CHWs play a vital role within public health systems by bridging the gap between health care services and communities through outreach, education, and advocacy [10]. Their contributions to health literacy promotion, counseling, and social support have demonstrated effectiveness across various chronic conditions [11]. In mental health care, CHWs are pivotal in the early intervention of conditions such as anxiety, depression, and trauma, potentially preventing the progression to more severe outcomes and reducing dependence on emergency services [12]. Moreover, their efforts empower individuals and communities to adopt proactive health management strategies [13].

Given their established contributions to mental health care, it is equally important to consider the expanding role of CHWs in cancer, a disease that represents one of the most significant global public health burdens, with markedly unequal effects across populations. In 2022, approximately 20 million new cancer cases and 9.7 million deaths were reported worldwide [14]. In terms of absolute burden, countries with a high Human Development Index (HDI) are projected to experience the largest increase in incidence, with an additional 4.8 million new cases expected by 2050, compared to 2022 estimates. However, the proportional increase in incidence is most notable in countries with a low HDI (142% increase) and in countries with a medium HDI (99%). Similarly, cancer mortality in these countries is projected to nearly double by 2050 [14].

CHWs are trusted intermediaries between marginalized populations and health systems, enabling their participation to significantly increase early detection and adherence to cancer screening (particularly breast, cervical, and colorectal cancers), as well as reduce inequities [15,16]. They also play a critical role in advanced care by fostering supportive care practices, reducing hospitalizations, and encouraging advance care planning. CHWs have also demonstrated long-term effectiveness in improving chronic disease management and reducing health disparities among minority populations [17,18,19]. In mental health support, their involvement is associated with sustainable improvements in health behaviors and overall community well-being [16]. Recently, CHWs have been integrated into cancer care, contributing to prevention efforts and survivorship support. Programs leveraging CHWs to deliver culturally tailored education on cancer screening have significantly increased screening rates among minority populations [20,21].

Beyond prevention, CHWs contribute to managing the physical and psychological burden associated with cancer. Individuals with cancer and survivors report poorer health status, increased distress levels, and a higher prevalence of chronic conditions compared to individuals without a cancer history [22]. Factors such as limited social support, low socioeconomic status, and persistent treatment-related symptoms further exacerbate vulnerability [23]. Involvement of CHWs in mental health interventions—such as psychoeducation, peer support groups, and referrals—has demonstrated improvements in emotional well-being and quality of life among cancer survivors [24].

Moreover, CHWs serve as essential intermediaries, helping patients navigate complex health care systems, largely through the provision of informational/educational, instrumental/logistical, affective/emotional, and appraisal support [25,26]. Despite these well-documented benefits, significant gaps remain regarding their effectiveness in palliative or supportive care settings [27]. This highlights the urgent need for targeted training on complex health topics, such as mental health in the context of cancer, which remains insufficient. Outreach and dissemination strategies must consider cultural factors to enhance CHW engagement and training efficacy [28].

### 1.2. The Importance of Training Lay Community Members as Mental Health Workers

Community-based participatory research (CBPR) emerges as a crucial framework for addressing the challenge of translating research findings into real-world practice, particularly within CHW programs [29]. CBPR acknowledges the expertise of community members in co-creating knowledge aimed at promoting public health and health equity [30]. By actively involving communities, CBPR facilitates the dissemination of scientific knowledge to marginalized populations, bridging critical access gaps [29].

One of the key ongoing challenges is preparing a workforce that is adequately trained to deliver psychological interventions grounded in evidence [31]. To support this goal, it is essential that training programs and curricula be culturally adapted, allowing CHWs to build the core competencies needed to engage with their communities effectively. This approach fosters mental health equity and helps broaden access to services. Furthermore, evaluations of these programs should consider not only clinical outcomes but also factors such as community empowerment and the long-term sustainability of the interventions [29].

Although the role of CHWs in mental health care is well established, research on the most effective training strategies remains limited. Recognizing this gap, the present study seeks to review, analyze, and synthesize the best practices identified across studies involving CBPR, CHWs, mental health, and cancer care. The overarching goal is to strengthen community capacity by equipping CHWs with essential training and resources to proactively address cancer care and related health conditions.

### 1.3. Study Aims and Objectives

Narrative reviews play a crucial role in advancing knowledge within a discipline by identifying, analyzing, evaluating, and interpreting the body of research on a specific topic. The present narrative review examines the educational and training strategies designed to equip CHWs with primary prevention skills in mental health. These competencies are essential for enabling CHWs to identify, manage, and refer individuals diagnosed with cancer, as well as their caregivers, who may be at an elevated risk for developing mental health disorders. The objectives of this review are to identify published articles that describe training protocols for CHWs, define the essential skills required in cancer and mental health contexts, examine the didactic strategies employed, and assess the scope and outcome of existing training programs. Our future plan is to use these findings to develop mental health training for community health workers in cancer care.

## 2. Materials and Methods

### 2.1. Study Design

This study was a narrative review conducted to identify key characteristics of training materials designed to equip lay community members as CHWs. The Covidence platform (Veritas Health Innovation, Melbourne, Australia) was utilized to facilitate the review process. Given the high volume of literature published annually on this topic, a selective approach was taken, prioritizing studies based on methodological rigor, relevance to the research objective, and contribution to understanding the essential components of training programs for CHWs [32,33]. To ensure appropriate expertise in information retrieval, the team received training and direct guidance from an academic librarian. However, resource limitations did not allow for a librarian to be a permanent member of the research team. In addition, several team members had prior experience in conducting literature reviews and designing search strategies.

Although PRISMA guidelines were not strictly applied, given the narrative nature of this review, a flowchart was included to enhance clarity and methodological transparency. This review was carried out as a narrative review rather than a systematic review, because no formal quality appraisal of the included studies was performed with standardized instruments. Instead, the selection of studies was guided by their methodological transparency, their relevance to the aims of the review, and the extent to which they contributed to understanding training strategies for CHWs. This approach made it possible to bring together essential elements from diverse sources, while recognizing that the absence of a structured critical appraisal represents a methodological limitation.

### 2.2. Eligibility Criteria

This narrative review included experimental studies such as randomized controlled trials (RCTs), non-randomized controlled trials (NRCTs), and quasi-experimental studies (pre-post designs) that described training components, strategies, or skills related to mental health prevention and promotion for CHWs. Systematic reviews and meta-analyses addressing CHW training components or competencies were also considered eligible. Studies targeting adults aged 18 years or older who were working as CHWs and describing mental health training programs or interventions were included. Web-based sources providing information on CHW training components, curricula, strategies, or educational materials were also screened. Interventions incorporating a mental health component and targeting mental health conditions (such as substance use, depression, anxiety, caregiver burden, and dementia), chronic conditions (such as diabetes, cardiovascular disease, and Human Immunodeficiency Virus [HIV]), and cancer were eligible. Only articles published in English or Spanish were considered for inclusion.

### 2.3. Search Strategy

A comprehensive search strategy was developed to retrieve relevant studies addressing the training of CHWs in mental health and cancer care. Searches were conducted through PubMed, EBSCOhost, Scielo, Redalyc, and Google Scholar, covering the period from 17 November 2023 to 7 March 2024. No restrictions were placed on the publication year to ensure a thorough capture of foundational and recent literature. The search utilized a series of keyword combinations aligned with the study’s four specific aims. Keywords included combinations such as “training OR teaching” AND “community health worker” AND “mental health” AND “cancer”; “training OR teaching OR capacity building” AND “community health worker” AND “mental health” AND “cancer”; and “curriculums OR protocols” AND “mental health promotion” AND “community health workers” to identify studies focusing on training components. To explore mental health prevention and promotion skills, combinations like “mental health prevention OR mental health promotion skills” AND “mental health workers OR lay health workers OR health promoters” and “mental health” AND “community mental health workers” AND “protocols AND cancer” were used. For identifying didactic approaches and training strategies, the search included “didactics OR strategies” AND “training” AND “community mental health workers” AND “cancer”. Finally, to capture training specifically related to cancer care, the search incorporated the keywords “community mental health workers” AND “mental health training” AND “cancer”. This structured approach was designed to maximize the retrieval of the relevant literature for this narrative review. Alongside peer-reviewed publications, we also examined web-based materials and non-academic sources, such as program manuals and organizational reports, to identify relevant training resources that are not typically indexed in academic databases.

### 2.4. Study Selection

All studies identified through the search strategy underwent an initial screening process based on titles and abstracts to assess adherence to the inclusion criteria. Studies were excluded if they focused exclusively on formally trained mental health professionals, such as clinical psychologists, social workers, or nurses, or if they involved participants under 18 years of age. In addition, studies lacking detailed descriptions of the training protocols or those presented solely as narrative reviews without new empirical data were excluded. After the preliminary screening, full-text articles were reviewed to ensure that they met all eligibility criteria before being included in the final analysis. One of the main challenges encountered was that many of the initially captured studies were clinical trials or intervention studies that focused on patient outcomes or opinions rather than describing CHW training initiatives. In addition, a considerable number of excluded records consisted of narrative reviews, conference abstracts, commentaries, or studies unrelated to mental health training for CHWs. These were screened out to ensure that only empirical studies or detailed descriptions of training programs were included in the final analysis. Studies were considered eligible if they described training initiatives for community health workers. These initiatives had to be aimed at improving skills—in general, mental health promotion and prevention. They also had to include resources to support people with chronic conditions such as diabetes, HIV, or cancer, who experience comorbid mental health issues.

### 2.5. Data Extraction and Management

Data from the included studies were systematically extracted and organized using an Excel spreadsheet, version 2508 (Microsoft Corporation, Redmond, WA, USA). For each study, relevant information such as study ID, title, authors, geographic region, study characteristics, methodologies employed, participant demographics, and inclusion and exclusion criteria were documented. Web-based resources and gray literature sources were similarly cataloged to maintain consistency across data sources. This structured approach facilitated efficient organization and enabled a comparative analysis across the selected articles, ensuring a comprehensive synthesis of the key components, strategies, and educational frameworks described for community mental health worker training programs.

### 2.6. Data Analysis

Data extracted from the selected studies were analyzed using a descriptive and thematic synthesis approach. Studies were organized into categories (e.g., research articles, training curricula, web-based resources, protocols) to facilitate systematic examination. An inductive process was applied to identify themes directly from the content of the included studies, rather than from a predetermined set of codes. To ensure consistency, eight members of the research team independently reviewed the extracted data and reached consensus through discussion when discrepancies arose.

## 3. Results

### 3.1. Article Selection Process

**Figure 1:**
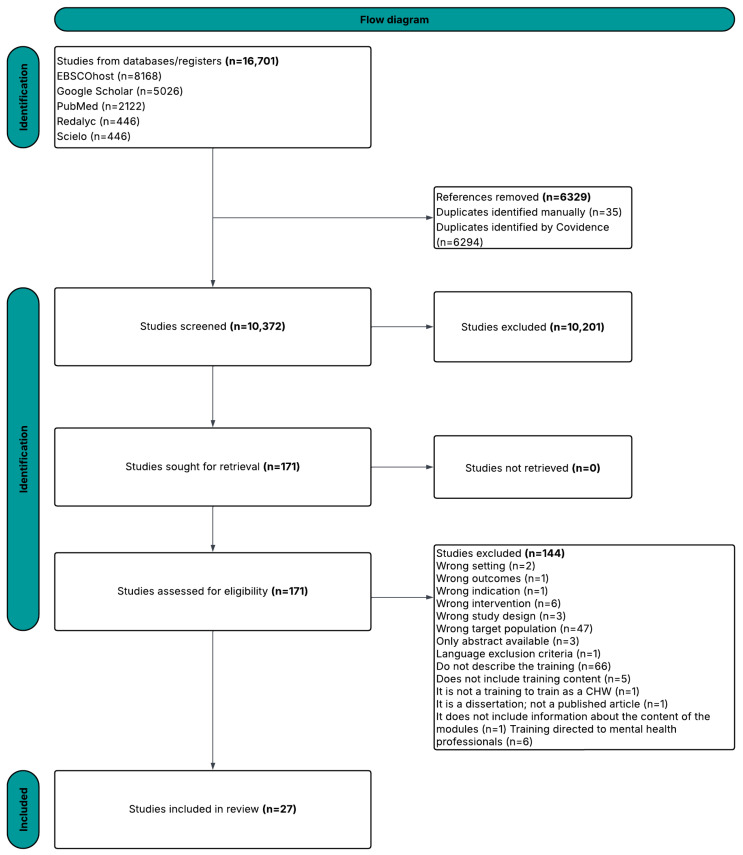
Flow diagram illustrating the procedure for selecting work for inclusion in our study.

A total of 16,701 publications were evaluated by independent reviewers using Covidence during the screening process (Figure 1). Of these, 6329 were identified as duplicates and removed. The remaining 10,372 references were considered potentially relevant and underwent title and abstract screening resulting in the exclusion of 10,201 articles that were not relevant to our study. A total of 171 articles proceeded to full-text review, of which 144 were excluded because they did not meet the inclusion criteria. Ultimately, 27 articles were included for comprehensive data extraction analysis. The main reasons for exclusion at the full-text stage were that many articles did not describe training components, focused on health professionals other than CHWs, or lacked empirical data. Additional records consisted of editorials or opinion pieces, which were also excluded. These were categorized as research articles (*n* = 16), training materials (*n* = 7), curricula (*n* = 1), and others (*n* = 3).

### 3.2. Training Components

In the 27 studies reviewed, we identified diverse training components for CHWs. These covered both mental health topics and different teaching strategies. Most studies (24 of 27) focused exclusively on mental health. This helped CHWs learn to recognize and address symptoms such as depression and anxiety, provide support, and refer people to medical care. Only three studies worked with populations facing chronic conditions, such as diabetes or HIV. These included content on mental health, but it was often treated as a secondary topic. In short, mental health and physical health were not addressed together and in a coordinated manner. Instead, they were kept separate. The most frequently reported component was mental health education, cited in 77.8% (*n* = 21) of the studies, emphasizing the foundational importance of disseminating knowledge in community-based mental health initiatives. Psychological intervention skill training was present in 44.4% (*n* = 12) of studies, often oriented toward equipping CHWs with practical tools for emotional support and behavioral management. A smaller subset (18.5%, *n* = 5) incorporated evidence-based intervention models, suggesting a growing, yet still limited, emphasis on standardized clinical approaches within community settings.

Additional training elements included family or caregiver-focused interventions (7.4%, *n* = 2), addressing health disparities (29.6%, *n* = 8), psychological first aid (22.2%, *n* = 6), and strategies to reduce mental health stigma (14.8%, *n* = 4). Moreover, 11.1% (*n* = 3) of studies provided content specifically focused on the role of CHWs in managing mental health issues, highlighting efforts to define and expand their scope of practice. Importantly, prevention strategies were addressed in 63.0% (*n* = 17) of the studies, indicating a strong emphasis on proactive care.

In terms of instructional methodology, instructional techniques were explicitly described in 33.3% (*n* = 9) of the studies. These included didactic sessions, interactive workshops, and experiential learning formats, although detailed reporting of teaching methods remained inconsistent across the literature. Together, these components underscore the heterogeneity of training approaches and content areas, while also revealing gaps in the systematic application of evidence-based models and instructional design.

### 3.3. Ethical and Pedagogical Approaches

This review highlighted various methods used to reinforce ethical principles and employ diverse teaching strategies in the training of CHWs. Ethical training commonly included content on research protections through CITI training, education on the Health Insurance Portability and Accountability Act (HIPAA), and instruction on the informed consent process both for CHWs during recruitment and for the individuals they serve.

Pedagogically, many programs incorporated didactic lectures, conferences, workshops, and classroom-based sessions, in addition to interactive tools such as games and case studies. Modeling strategies, particularly role-playing and the use of training manuals, were frequently employed to enhance engagement and learning outcomes. Audiovisual materials and educational aids were also used to support dynamic learning and accommodate various learning styles.

### 3.4. Instructional Strategies and Tools

The reviewed literature revealed a wide array of didactic approaches. The most frequently reported were role-playing (*n* = 5), videos (*n* = 4), manuals (*n* = 4), and presentations using PowerPoint, flipcharts, and brochures (*n* = 2). Other instructional methods included graphics and illustrations (*n* = 2), lectures (*n* = 2), case studies (*n* = 2), group work (*n* = 2), didactics (*n* = 2), supervision (*n* = 2), and field training (*n* = 2). Less commonly reported strategies involved virtual learning platforms such as Zoom (*n* = 1) and learning management systems like Canvas or Moodle (*n* = 1). Additional innovative methods included peer learning through pair activities, traditional classroom settings, games, case vignettes, software applications, video-based feedback, skills practice, and brainstorming—each of which was reported in one study.

### 3.5. Characteristics of Training Programs for Community Health Workers

#### 3.5.1. Aim 1: Description of Training Components

The literature review revealed a range of materials implemented in training initiatives. These included research materials (59.3%, *n* = 16), training manuals or guides (25.9%, *n* = 7), curriculum documents (3.7%, *n* = 1), and other materials (11.1%, *n* = 3), such as program descriptions, training evaluations, and academic theses. Research materials were the most frequently utilized, followed by general training documents, with fewer instances of structured curricula or alternative formats.

Training programs were generally comprehensive and structured to provide CHWs with essential skills for supporting individuals with mental health needs in diverse contexts. Various training programs included components focused on crisis intervention, offering strategies for managing acute psychological symptoms such as suicidal ideation, panic attacks, and intense emotional distress. Case management training was also frequently reported and typically covered assessment, referral pathways, and follow-up procedures to maintain continuity of care. Programs placed a strong emphasis on the development of practical skills, utilizing techniques such as role-playing, case study analysis, and structured exercises in motivational interviewing, behavioral activation, and problem-solving.

Moreover, many programs integrated community engagement strategies, aiming to enhance mental health promotion, reduce stigma, and mobilize community-based resources. Administrative elements, including the refinement of training materials and the implementation of supervision and feedback systems, were also present to support training quality and worker preparedness. Overall, these components are not only foundational for theoretical understanding but are also critical in empowering CHWs to respond effectively to the complex psychosocial realities of the communities they serve.

#### 3.5.2. Aim 2: Mental Health Prevention and Promotion Skills

Training programs for CHWs emphasize the development of a broad set of practical skills essential for delivering effective mental health support. A key foundational component across the reviewed programs was the ability to build and maintain supportive, trust-based relationships with clients. They also emphasize recognizing and addressing various mental health issues, as well as navigating mental health service systems effectively. CHWs are trained to engage empathetically, set achievable goals, and negotiate service agreements. They are also equipped to advocate for clients, mobilize community resources, and facilitate timely referrals when necessary. Skills such as understanding and applying crisis intervention techniques, managing suicide risk, and providing psychological first aid are integral parts of the training. Furthermore, CHWs are prepared to respond to various mental health emergencies, including identifying and addressing suicidal ideation, de-escalating panic attacks, and offering guidance following traumatic events. CHWs need core skills to effectively address mental health needs in their communities. High-quality training emphasizes client engagement, recognizing mental health concerns, navigating public and private services, and advocating for clients. These skills enable CHWs to build trust, deliver appropriate care, and empower community members to access available resources.

Training highlights the importance of the development of crisis intervention and mental health education, equipping CHWs to handle acute situations and promote mental well-being. Through the development of these skills, CHWs are better positioned to deliver comprehensive, empathetic, and culturally relevant mental health support to individuals and families in their communities.

#### 3.5.3. Aim 3: Training Strategies for Community Health Workers

An analysis of the selected studies reveals that in-person training was the predominant method used for CHW instruction, accounting for 63.0% (*n* = 17) of the identified strategies. This modality was favored for its ability to foster the development of practical skills, encourage direct interaction, and provide immediate feedback elements deemed critical for CHWs working in community settings. In contrast, hybrid training approaches (combining in-person and online modalities) were reported in only 3.7% (*n* = 1), suggesting limited adoption despite their potential for flexibility and scalability.

Additionally, 22.2% (*n* = 6) of the studies did not specify the method of training delivery, indicating a gap in reporting and standardization across programs. Meanwhile, 11.1% (*n* = 3) utilized two or more delivery methods, highlighting the potential benefits of blended learning environments that draw on multiple instructional techniques. Of the three studies that reported the use of two or more instructional methods (11.1%), none were designed as comparative trials contrasting single versus multimodal approaches. Rather, the integration of strategies (e.g., integrating role-playing with manuals or video content) was used as a practical resource to increase participation, address different learning styles, and enhance knowledge retention. Therefore, the potential benefits are based on observations of practice and experience, not on comparative studies. The overall predominance of face-to-face formats underscores a preference for immersive, hands-on training that facilitates CHWs engagement and skill development within the communities they serve.

Didactic strategies in CHW training programs commonly included the use of manuals, presentations, graphics, illustrations, and video-based content. Six studies specifically referenced the use of manualized materials, which were adapted to various contexts and delivery formats [34,35,36,37,38,39]. For example, Chiumento et al. [34] adapted an individual intervention manual for group settings, incorporating content on psychoeducation, motivational interviewing, stress management, behavioral activation, and social support enhancement.

Didactic presentations were another frequently reported strategy, used to structure and deliver core content [37,38,40,41,42]. Uriarte et al. [40] described the use of slide-based curricula designed to scaffold complex content and avoid cognitive overload. Graphics and illustrations were employed to enhance accessibility and visual learning, as supported by Glenberg and Langston [43], who argued that visual aids help readers build mental models and improve comprehension [9,36,38,40,41].

Video content also played a prominent role in training programs [35,38,44,45,46]. Tyagi et al. [38] partnered with a local video production company to create a short, cultural platform accessible via smartphones and tablets.

Role-playing exercises were widely used across studies to develop communication skills and practical competencies [9,12,35,37,40,42,45,46]. These exercises were applied in various contexts, including mental health screening, cancer care communication, HIV interventions, and leadership development. For instance, within the MESA intervention, role-play activities helped participants apply coping strategies in simulated real-life scenarios, reinforcing both content knowledge and experiential learning.

Additional strategies included modeling, interactive lectures, group discussions, supervision site visits, and pair activities, as reported by Bordeaux-Rank [47], Shields-Zeeman et al. [45], and Uriarte et al. [40]. These methods facilitated knowledge retention and skill acquisition through dynamic and participatory leaning environments. For example, Moore-Monroy et al. [41] integrated tools such as Flip *n* Tell easel binders, anatomy visuals, and localized community resources to contextualize learning within structural health care barriers.

Beyond simulated exercises, several training programs incorporated real-word fieldwork to reinforce knowledge application. Malla et al. [44] emphasized clinical shadowing and hands-on sessions in outpatient settings, with instruction on risk management protocols and patient documentation. Similarly, Monton et al. [46] implemented a three-month curriculum combining synchronous and asynchronous learning with experiential field training, aiming to enhance CHWs’ competencies in palliative care.

Bordeaux-Rank [47] developed a five-day Training of Trainers program based on the Community-Based Psychological First Aid (CBPFA) model. This model emphasized community resilience and psychological support during emergencies. The training included interactive lectures and field-based activities, equipping CHWs with practical tools to deliver psychological first aid and make timely referrals for professional intervention.

### 3.6. Overview of Training Content and Structure

A synthesis of selected trainings interventions reveals a range of methodologies, implementation contexts, and target populations that illustrate the diverse applications of community-based mental health training programs (see Table 1). The summarized studies include quasi-experimental, qualitative, and mixed methods designs, implemented across diverse regions such as India, South Africa, and the United States. Target populations included rural CHWs, direct care personnel, and volunteer community educators, all of whom were engaged in strengthening local mental health capacities.

Regarding training content, key components included evidence-based intervention models, instructional techniques, mental health education, and psychosocial interventions skills (see Table 2). Several programs also addressed prevention, stigma reduction, and health disparities. For instance, Taking Charge of My Life and Health focused on personal empowerment and self-care, while initiatives such as MESA and SONRISA offered culturally tailored approaches for promotoras and migrant communities. These trainings reflect a multidimensional framework aimed at building technical, psychoeducational, and community-oriented competencies among CHWs and other frontline actors.

Overall, a comparison across interventions shows that programs combining didactic content with interactive strategies such as role-playing, case discussions, or field-based practice tended to report stronger improvements in CHWs’ knowledge, confidence, and communication skills. In contrast, training relying primarily on didactic lectures or printed manuals provided more limited evidence of sustained outcomes. This comparative perspective highlights the added value of experiential and participatory methods for strengthening CHWs’ mental health competencies.

## 4. Discussion

### 4.1. Summary of Findings Regarding Training Characteristics

The findings of this narrative review reveal that CHWs’ training programs are remarkably comprehensive and integrate a wide range of essential components for providing effective mental health support. This is evidenced by previous research highlighting the need for holistic training that includes theoretical foundations and practical tools for addressing community mental health challenges. The continued incorporation of research materials as core components in training design highlights a growing emphasis on evidence-based approaches. This is consistent with existing research advocating for the development of data-driven training to ensure effectiveness and contextual relevance.

The key components of the training identified, such as the detection of mental health needs in diverse populations, crisis intervention strategies (e.g., for suicidal ideation and panic attacks), and case management, reflect standard best practices reported in the community health literature. Additionally, the use of interactive methods like role-playing, case studies, and motivational interviewing coincides with findings from previous studies that suggest experiential learning improves skill acquisition and confidence among CHWs.

When comparing across studies, several patterns emerged. While many programs relied heavily on didactic lectures or printed manuals, interventions that combined didactic content with interactive strategies, such as role-playing, case discussions, or field-based practice tended to report stronger improvements in CHWs’ knowledge, confidence, and communication skills. This highlights the heterogeneity of approaches used across the literature and points to persistent gaps in the systematic use of evidence-based instructional models. These findings suggest that experiential and participatory methods may be especially valuable for strengthening CHWs’ mental health competencies and should be further prioritized in the design of future training initiatives.

### 4.2. Implications for Community Health Workers’ Training Programs

These results have significant implications for the structure and delivery of training programs for community health workers. By integrating a broad range of didactic and practical elements, the programs ensure that community health workers are prepared to address challenging mental health needs in their communities. Therefore, the focus on cultural competency is particularly relevant in multicultural contexts. This echoes previous research, which highlights the need for training that promotes culturally responsive care. This component will not only strengthen the therapeutic alliance but also help promote the accessibility and equity of mental health services.

The emphasis on community engagement, stigma reduction, and resource mobilization indicates a paradigm shift towards more proactive and community-centered models of mental health care. Furthermore, the attention to supervision, feedback mechanisms, and administrative refinement suggests a commitment to continuous quality improvement, which is essential for adapting to evolving community needs and maintaining the efficacy of interventions.

These findings are particularly relevant in settings affected by concurrent social and environmental stressors. One of the aims of this review was to support the design of training programs that strengthen the ability of CHWs to work effectively in settings where structural challenges amplify mental health needs. For example, in Puerto Rico following Hurricane Maria, medically vulnerable populations, such as cancer survivors, have faced additional barriers to access to care. This highlights the importance of training programs that prepare CHWs to respond effectively to crises and mental health needs within fragile infrastructures [52,53]. This is why addressing these vulnerabilities in training content ensures that community health workers are equipped to provide emotional support and community resilience during emergencies.

Taken together, these results suggest that future training efforts for CHWs should be guided by a structured framework that combines three key elements. First, culturally adapted didactic and interactive strategies such as role playing, case-based exercises, and audiovisuals tools appear to be more effective than lectures or manuals alone. Second, evaluation should not be limited to knowledge gains, but also include changes in confidence, attitudes, and community-level outcomes. Third, implementation factors such as supervision, ongoing support, and scalability through hybrid or digital platforms must be considered to ensure sustainability. Framing CHW training programs within these dimensions may enhance their impact and promote the integration of mental health support into cancer care.

### 4.3. Gaps in Cancer-Related CHW Training Programs

According to the findings and scope of this narrative review, there is a notable lack of published information on formal training programs specifically designed for CHWs that address the mental health needs of cancer patients, survivors, caregivers, and those at risk of psychological distress related to cancer. This gap must be addressed to develop and establish training programs capable of providing the necessary tools to meet the community-level needs of these groups. Training programs in mental health and cancer are essential for educating health professionals and communities on a comprehensive approach to these diseases. Such programs should include psycho-oncology, intervention techniques, stress management, and emotional support, enabling holistic care that enhances the quality of life for patients and their families. Additionally, they should promote interdisciplinary collaboration and a deeper understanding of how cancer and its treatment impact mental health, ensuring that professionals and CHWs are equipped to provide empathetic and effective palliative care to patients with cancer and their families.

### 4.4. Future Directions for Research and Practice

This review lays the groundwork for subsequent phases of the project, particularly the joint development of training materials. These are culturally adapted in collaboration with a Community Intervention Team. Future research should aim to evaluate the effectiveness of these training programs once implemented, including longitudinal evaluations of CHW performance, service delivery outcomes, and community mental health indicators. Furthermore, additional studies are needed to investigate the scalability and sustainability of these training models in diverse geographic and sociocultural contexts.

Practical examples, such as the Women’s Holistic Health (Salud Holística para la Mujer, SAHOM) program and the peer-led Nuevo Amanecer-II intervention, underscore the value of culturally tailored initiatives based on community-identified needs. Although SAHOM is not a training program per se, its participatory development process and holistic emphasis on emotional well-being illustrate how principles of cultural equity and community responsiveness can inform effective interventions. In contrast, the peer-led Nuevo Amanecer-II demonstrated the potential for integrating experiential learning, empathy, and sociocultural awareness into community-based psychosocial support models [54,55]. Future research should explore how such community-centered frameworks could be adapted and expanded to enhance the work of community health workers.

In addition to these cultural and participatory dimensions, this literature review also highlights a technological gap. Our analysis of 27 studies published between 1987 and 2023, showed that while training approaches have evolved from traditional and manualized methods toward digital formats in recent years (e.g., [56,57]), none incorporated artificial intelligence (AI), explainable AI (XAI), or intelligent training platforms. This absence may be explained by the relatively recent development of such technologies. Identifying this gap is crucial, as future research should examine how AI-driven tools, such as conversational agents, mobile-based screening, and adaptive e-learning platforms, might complement culturally tailored models, enhance individual adaptation, and support the long-term sustainability of CHW training programs in mental health.

## 5. Conclusions

### 5.1. Key Takeaways from the Narrative Review

The literature review provides insight into specific areas and topics, while highlighting knowledge gaps that remain to be explored [58]. A key finding of this narrative review was the identification of a substantial gap in the availability of training programs specifically designed to prepare CHWs to support people diagnosed with cancer and their caregivers. These programs should include components of prevention, assessment, identification, treatment, and referral to specialized care when necessary.

The review also identified and classified other training programs aimed at addressing chronic conditions such as diabetes, obesity, anxiety, and depression, among others. Additionally, it analyzed the most employed didactic strategies and assessed their frequency of implementation. According to our findings, the most used role-plays, instructional manuals, and didactic presentations were important. This lays the groundwork for future studies to determine which strategies are most appropriate for training health promoters in mental health and chronic conditions, such as cancer.

### 5.2. Importance of Ongoing Efforts in Training Community Health Workers

The importance of continuing efforts to train CHWs is underscored by current statistics and projections, which estimate that over 39.5% of the population will experience cancer, a condition that has both physical and mental health consequences [59]. There is a well-documented need for increased access to mental health services in both the United States and Puerto Rico. Given that Puerto Rico is frequently impacted by atmospheric events, seismic activity, and systemic challenges in health care infrastructure, it is crucial to implement strategies that empower communities to manage prevention, identification, treatment, and referral processes effectively.

### 5.3. Call for Further Research and Program Development

This narrative review enabled a rigorous and comprehensive exploration of the existing literature and revealed a clear gap in the development of training programs and research focused on CHWs who support individuals with comorbid cancer and mental health conditions, as well as their caregivers. This review offers a foundation for future projects aiming to design training models grounded in evidence-based recommendations and pedagogical strategies tailored to this specific context, with cultural sensitivity to the Puerto Rican population.

Considering Puerto Rico’s current epidemiological and sociopolitical context, it is important that professionals in psychology, public health, medicine, and related disciplines collaborate in the development of culturally and contextually appropriate programs. These initiatives should have the primary objective of empowering communities with the tools and resources necessary to respond autonomously and effectively to their specific health challenges, especially considering the limitations of traditional treatment and referral systems, which are often inaccessible to the population.

## Figures and Tables

**Table 1:** Summary of selected community-based mental health training programs.

Author (Year)	Study Design	Country	Target Audience	Key Theme
Armstrong et al. (2011) [9]	Quasi-experimental	India	Rural Community Health Workers	Mental health training for CHWs
Bordeaux-Rank (2014) [47]	Mixed methods	U.S. (Native American communities)	Direct Care Staff	Community-based psychological first aid
Chaudhary et al. (2019) [48]	Cohort study	U.S. (Syrian refugees)	Volunteer Health Educators	Cultural integration and health access
Sibeko et al. (2018) [49]	Mixed methods	South Africa	Community Health Workers	Knowledge, confidence, attitudes shift
Tyagi et al. (2023) [38]	Qualitative	India	Rural Community Health Workers	Digital training on schizophrenia detection

Note: Detailed information on all reviewed training programs, including full methodology, population characteristics, and comprehensive content descriptions, is available in Appendix A (Table A1).

**Table 2:** Summary of key skills categories in reviewed training programs.

Article (Year)	Training Name	Key Skill Categories
Abadi et al. (2020) [50]	Taking Charge of My Life and Health	Evidence-Based Intervention Models; Roles of CHWs in mental health; Psychological intervention skills; Prevention
Bordeaux-Rank (2014) [47]	Community-Based Psychological First Aid	Instructional Techniques; Psychological Intervention Skills
Edelblute et al. (2014) [42]	MESA (Mujeres en Solidaridad Apoyándose)	Instructional Techniques; Psychological Intervention Skills; Prevention
Reinschmidt & Chong (2005) [51]	SONRISA	Evidence-Based Intervention Models; Roles of CHWs; Mental Health Education; Prevention
Tyagi et al. (2023) [38]	Digital Training Program (COPSI-based)	Instructional Techniques; Mental Health Education; Stigma Reduction; Health Disparities Reduction

Note: Detailed information on all reviewed training programs, including full methodology, population characteristics, and comprehensive content descriptions, is available in Appendix A (Table A2).

## Data Availability

This study is a narrative literature review. No new data were generated or analyzed in the course of this research. However, details regarding the search strategy, including the databases consulted and the selection process conducted via COVIDENCE, are available upon reasonable request. Interested researchers may contact the corresponding author for further information.

## Abbreviations

The following abbreviations are used in this manuscript:

CHWs	Community Health Workers
CBPR	Community-based participatory Research
RCTs	Randomized Controlled Trials
NRCTs	Non-Randomized Controlled Trials
HIV	Human Immunodeficiency Virus
CBPFA	Community-based Psychological First Aid
HIPPA	Health Insurance Portability and Accountability Act
SAHOM	Salud Holística para la Mujer

## Appendix A

**Table A1:** Selected community-based mental health training programs.

Authors	Title	Year	Methodology/Study Desing	Target Audience	Country
Abadi, M. H., Drake, C., Richard, B. O., Schweinhart, A., Rychener, D., Shamblen, S. R., & Grimsgaard, S. N. [50]	An evaluation of the facilitator training to implement ‘Taking charge of my life and health’, a peer-led group program to promote self-care and patient empowerment in Veteran participants	2020	Quasi-experimental	Veteran peers	United States
Andreae, S. J., Andreae, L. J., Cherrington, A. L., Lewis, M., Johnson, E., Clark, D., & Safford, M. M. [60]	Development of a Community Health Worker-Delivered Cognitive Behavioral Training Intervention for Individuals with Diabetes and Chronic Pain	2018	Qualitative	Ten community members were trained and certified as CHWs. All were females, nine were African American, all had some college or a college degree, and five reported an income of USD 40,000 or more per year	United States
Armstrong, G., Kermode, M., Raja, S., Suja, S., Chandra, P., & Jorm, A. F. [9]	A mental health training program for community health workers in India: impact on knowledge and attitudes	2011	Quasi-experimental	Training participants were community health workers sourced through Gramina Abrudaya Seva Samstha (GASS), an NGO operating in Doddaballapur Taluk, Bangalore Rural District, Karnataka, India. The types of community health workers included Junior Health Assistants, Village Rehabilitation Workers, and ASHA workers	India
Bordeaux-Rank, J. A. [47]	Tribal Participatory Research with Community-based Psychological First Aid Training for Native Direct Care Staff: A Continuation Study	2014	Mixed Methods	Native American communities	United States
Chaudhary, A., Dosto, N., Hill, R., Lehmijoki-Gardner, M., Sharp, P., Daniel Hale, W., & Galiatsatos, P. [48]	Community Intervention for Syrian Refugees in Baltimore City: The Lay Health Educator Program at a Local Mosque	2019	Cohort study	Community members who would volunteer their time to learn about health care resources and information that could be utilized to aid refugees in their assimilation into the US healthcare system	United States
Cheffi, A., Harrison, S., & Stephan, A. [61]	Community Based Mental Health–A Training Guide for Community Providers	2023	Does not describe		Does not describe
Edelblute, H. B., Clark, S., Mann, L., McKenney, K. M., Bischof, J. J., & Kistler, C. [42]	Promotoras across the border: a pilot study addressing depression in Mexican women impacted by migration	2014	Quasi-experimental	Mexican immigrant women in the US	Mexico
Gormley, M., Loughran, C., Conte, J., & Dunn Navarra, A. M. [35]	Trends in U.S. HIV Peer Health Worker Training Strategies and Approaches: A Scoping Review of the Literature	2023	Scoping Review	U.S. HIV peer health workers	United States
Hofmann-Broussard, C., Armstrong, G., Boschen, M. J., & Somasundaram, K. V. [62]	A mental health training program for community health workers in India: Impact on recognition of mental disorders, stigmatizing attitudes and confidence.	2016	Experimental/non-randomized controlled trial	Rural India	India
Hossain, D., Gorman, D., Eley, R., & Coutts, J [63]	Farm Advisors’ reflections on Mental Health First Aid training	2009	Quasi-experimental	Thirty-two farm advisors working in Southern Queensland, Australia, who attended MHFA training	Australia
Malla, A., Margoob, M., Iyer, S., Joober, R., Lal, S., Thara, R., Mushtaq, H., & Mansouri, B. I. [44]	A Model of Mental Health Care Involving Trained Lay Health Workers for Treatment of Major Mental Disorders Among Youth in a Conflict-Ridden, Low-Middle Income Environment: Part I Adaptation and Implementation	2019	Qualitative	Recruited and trained 40 LHWs (18 males and 22 females) from villages within the Ganderbal district	India
McCabe, O. L., Marum, F., Mosley, A., Gwon, H. S., Langlieb, A., Everly, G. S., Jr, Kaminsky, M. J., & Links, J. M. [64]	Community capacity-building in disaster mental health resilience: a pilot study of an academic/faith partnership model	2012	Mixed Methods	Faith communities, participants representing Christian, Jewish, and Muslim FBOs, respectively	United States
Michael, A., Thirumoorthy, A., Girish, N., & Sivakumar, P. T. [65]	Training Community health workers in geriatric mental health: Process of manual development and pilot testing findings	2018	Quasi-experimental	Interviews (*n* = 23) used a semi-structured interview guide prepared by the researchers, and the study included geriatric mental health professionals, experts from non-governmental organizations, primary health center physicians, Taluk Officers, CHWs, community members, and the elderly. The semi structured questionnaire used was different for each group of key informants. Five health workers from one of the primary health centers under the BBMP south were the participants in the pilot. The Mental Health Literacy Survey Questionnaire was used to assess the difference between pre- and post-assessments	India
Paramasivam, R., Elangovan, A. R., Amudhan, S., Kommu, J. V. S., Haridas, H., & Sriramalu, S. B. [66]	Intervention-based mental health training for community level workers in India—A systematic review	2022	Systematic Review	CHWs and mental health	India
Quinnett P. G. [56]	The Certified QPR Pathfinder Training Program: A Description of a Novel Public Health Gatekeeper Training Program to Mitigate Suicidal Ideation and Suicide Deaths	2023	Qualitative	Person with a high risk of suicide	United States
Reinschmidt, K. M., & Chong, J. [51]	SONRISA: a curriculum toolbox for promotores to address mental health and diabetes.	2005	Qualitative	Promotores (community health workers) who work with Hispanic clients to prevent or manage diabetes	Latin America United States
Reinschmidt, K. M., Philip, T. J., Alhay, Z. A., Braxton, T., & Jennings, L. A. [57]	Training Community Health Workers to Address Disparities in Dementia Care: A Case Study from Oklahoma With National Implications	2023	Quasi-experimental	Older population in Oklahoma City	United States
Robertson, M. N., DeShong, H. L., Steen, J. S., Buys, D. R., & Nadorff, M. R. [67]	Mental health first aid training for Extension agents in rural communities	2021	Mixed Methods	Rural communities, farmers	United States
Ryan, A. S., & Epstein, I. [68]	Mental health training for Southeast Asian refugee resettlement workers	1987	Mixed Methods	Southeast Asian workers, refugees, refugee community support network in northeastern USA.	United States
Shields-Zeeman, L., Pathare, S., Walters, B. H., Kapadia-Kundu, N., & Joag, K. [45]	Promoting wellbeing and improving access to mental health care through community champions in rural India: the Atmiyata intervention approach	2017	Mixed Methods	Two populations experiencing varying levels of distress: people with emotional stress and/or common mental health problems and people with severe mental illness	India
Sibeko, G., Milligan, P. D., Roelofse, M., Molefe, L., Jonker, D., Ipser, J., Lund, C., & Stein, D. J. [49]	Piloting a mental health training programme for community health workers in South Africa: an exploration of changes in knowledge, confidence and attitudes	2018	Mixed Methods	Community health workers	South Africa
Smith, M. V., & Kruse-Austin, A. [13]	A gender-informed model to train community health workers in maternal mental health	2015	Quasi-experimental	Mothers living in the city of New Haven	United States
Stacciarini, J. M., Rosa, A., Ortiz, M., Munari, D. B., Uicab, G., & Balam, M. [69]	Promotoras in mental health: a review of English, Spanish, and Portuguese literature	2012	Mixed Methods	Community health workers	Western Countries
Tyagi, V., Khan, A., Siddiqui, S., Kakra Abhilashi, M., Dhurve, P., Tugnawat, D., Bhan, A., & Naslund, J. A. [38]	Development of a Digital Program for Training Community Health Workers in the Detection and Referral of Schizophrenia in Rural India	2023	Qualitative	Rural India	India
Weaver, A., & Lapidos, A. [70]	Mental Health Interventions with Community Health Workers in the United States: A Systematic Review	2018	Systematic Review	Systematic review of mental health interventions with CHWs in the United States	United States
Wennerstrom, A., Vannoy, S. D., 3rd, Allen, C. E., Meyers, D., O’Toole, E., Wells, K. B., & Springgate, B. F. [12]	Community-based participatory development of a community health worker mental health outreach role to extend collaborative care in post-Katrina New Orleans	2011	Qualitative	Community to address post-disaster mental health disparities	United States
Wright, J., & Chiwandira, C. [71]	Building capacity for community mental health care in rural Malawi: Findings from a district-wide task-sharing intervention with village-based health workers.	2016	Qualitative	Zomba district of southern Malawi. Consists of villages of 200–1000 people, small trading centers, and one urban center in Zombatown. The HSAs’ brief biomedical training and accessibility within local populations mean they have experience navigating differing perspectives of causation of and treatment for health problems	Malawi

**Table A2:** Key skill categories in reviewed training programs.

Reference of Article	Training Name	Training Skills Categories
Abadi, M. H., Drake, C., Richard, B. O., Schweinhart, A., Rychener, D., Shamblen, S. R., & Grimsgaard, S. N. (2020). An evaluation of the facilitator training to implement ‘Taking charge of my life and health’, a peer-led group program to promote self-care and patient empowerment in Veteran participants. *Patient education and counseling*, S0738-3991(20)30332-3. Advance online publication. https://doi.org/10.1016/j.pec.2020.06.014 [50]	“Taking charge of my life and Health”—a peer-led program	Evidence-based intervention models, roles of CMHWs in helping to prevent or manage mental health issues, psychological intervention skills training, prevention
Andreae, S. J., Andreae, L. J., Cherrington, A. L., Lewis, M., Johnson, E., Clark, D., & Safford, M. M. (2018). Development of a Community Health Worker-Delivered Cognitive Behavioral Training Intervention for Individuals With Diabetes and Chronic Pain. *Family & community* *health*, *41*(3), 178–184. https://doi.org/10.1097/FCH.0000000000000197 [60]	Does not describe the name of the training/intervention	Roles of CMHWs in helping to prevent or manage mental health issues
Armstrong, G., Kermode, M., Raja, S., Suja, S., Chandra, P., & Jorm, A. F. (2011). A mental health training program for community health workers in India: impact on knowledge and attitudes. *International journal of mental health systems*, *5*(1), 17. https://doi.org/10.1186/1752-4458-5-17 [9]	Does not describe the name of the training/intervention	Instructional techniques, mental health education, prevention, psychological first aid
Bordeaux-Rank, J. A. (2014). Tribal participatory research with community-based psychological first aid training for Native direct care staff: A continuation study. ProQuest Dissertations Publishing. [47]	Community-based psychological first aid training	Instructional techniques; psychological intervention skills training
Chaudhary, A., Dosto, N., Hill, R., Lehmijoki-Gardner, M., Sharp, P., Daniel Hale, W., & Galiatsatos, P. (2019). Community Intervention for Syrian Refugees in Baltimore City: The Lay Health Educator Program at a Local Mosque. *Journal of religion and health*, *58*(5), 1687–1697. https://doi.org/10.1007/s10943-019-00893-9 [48]	The Lay Health Educator Program Curriculum	Mental health education; prevention
Cheffi, A., & Harrison, S. (2023). Community-based mental health (CBMH) training guide. IFRC Reference Centre for Psychosocial Support. Copenhagen, Denmark. [61]	Community-Based Mental Health a Training Guide for Community Providers	Instructional techniques, mental health education, prevention, training aimed at reducing health disparities
Edelblute, H. B., Clark, S., Mann, L., McKenney, K. M., Bischof, J. J., & Kistler, C. (2014). Promotoras across the border: a pilot study addressing depression in Mexican women impacted by migration. *Journal of immigrant and minority health*, *16*(3), 492–500. https://doi.org/10.1007/s10903-012-9765-5 [42]	Mujeres en Solidaridad Apoyándose (MESA)	Instructional techniques, psychological intervention skills training, prevention
McCabe, O. L., Marum, F., Mosley, A., Gwon, H. S., Langlieb, A., Everly, G. S., Jr., Kaminsky, M. J., & Links, J. M. (2012). Community capacity-building in disaster mental health resilience: A pilot study of an academic/faith partnership model. International Journal of Emergency Mental Health, 14(2), 112–122. [64]	Does not describe the name of the training/intervention	Mental health education, psychological intervention skills training
Gormley, M., Loughran, C., Conte, J., & Dunn Navarra, A. M. (2023). Trends in U.S. HIV Peer Health Worker Training Strategies and Approaches: A Scoping Review of the Literature. *The Journal of the Association of Nurses in AIDS Care: JANAC*, *34*(4), 331–348. https://doi.org/10.1097/JNC.0000000000000415 [35]	Does not describe the name of the training/intervention	Prevention; instructional techniques
Hofmann-Broussard, C., Armstrong, G., Boschen, M. J., & Somasundaram, K. V. (2016). A mental health training program for community health workers in India: impact on recognition of mental disorders, stigmatizing attitudes and confidence. International Journal of Culture and Mental Health, 10(1), 62–74. https://doi.org/10.1080/17542863.2016.1259340 [62]	Does not describe the name of the training/intervention	Mental health education, training aimed at reducing health disparities, training aimed at reducing stigma
Hossain, D., Gorman, D., Eley, R., & Coutts, J. (2009). Farm Advisors’ reflections on Mental Health First Aid training. Australian E-Journal for the Advancement of Mental Health, 8(1), 105–111. https://doi.org/10.5172/jamh.8.1.105 [63]	Mental health first aid training	Prevention; instructional techniques; mental health education
Malla, A., Margoob, M., Iyer, S., Joober, R., Lal, S., Thara, R., Mushtaq, H., & Mansouri, B. I. (2019). A Model of Mental Health Care Involving Trained Lay Health Workers for Treatment of Major Mental Disorders Among Youth in a Conflict-Ridden, Low-Middle Income Environment: Part I Adaptation and Implementation. *Canadian journal of psychiatry. Revue canadienne de psychiatrie*, *64*(9), 621–629. https://doi.org/10.1177/0706743719839318 [44]	Does not describe the name of the training/intervention	Prevention; Instructional techniques; mental health education
Michael, A., Thirumoorthy, A., Girish, N., & Sivakumar, P. T. (2018). Training Community health workers in geriatric mental health: Process of manual development and pilot testing findings. *Asian journal of psychiatry*, *38*, 12–15. https://doi.org/10.1016/j.ajp.2018.10.017 [65]	Does not describe the name of the training/intervention	Prevention
Paramasivam, R., Elangovan, A. R., Amudhan, S., Kommu, J. V. S., Haridas, H., & Sriramalu, S. B. (2022). Intervention-based mental health training for community level workers in India -A systematic review. *Journal of family medicine and primary care*, *11*(4), 1237–1243. https://doi.org/10.4103/jfmpc.jfmpc_1134_21 [66]	Does not describe the name of the training/intervention	Mental health education; prevention; psychological intervention skills training; psychological first aids
Quinnett P. G. (2023). The Certified QPR Pathfinder Training Program: A Description of a Novel Public Health Gatekeeper Training Program to Mitigate Suicidal Ideation and Suicide Deaths. *Journal of prevention (2022)*, *44*(6), 813–824. https://doi.org/10.1007/s10935-023-00748-w [56]	Pathfinder QPR Gatekeeper Training Program	Prevention; mental health education; psychological first aid
Reinschmidt, K. M., & Chong, J. (2005). SONRISA: a curriculum toolbox for promotores to address mental health and diabetes. *Preventing chronic disease*, *4*(4), A101. [51]	Sonrisa	Evidence-based intervention models; roles of CMHWs in helping to prevent or manage mental health issues; mental health education; prevention.
Reinschmidt, K. M., Philip, T. J., Alhay, Z. A., Braxton, T., & Jennings, L. A. (2023). Training Community Health Workers to Address Disparities in Dementia Care: A Case Study From Oklahoma With National Implications. *The Journal of ambulatory care management*, *46*(4), 272–283. https://doi.org/10.1097/JAC.0000000000000470 [57]	Dementia Training for CHW	Mental health education; training aimed at reducing health disparities
Robertson, M. N., DeShong, H. L., Steen, J. S., Buys, D. R., & Nadorff, M. R. (2021). Mental health first aid training for Extension agents in rural communities. *Suicide & life-threatening* *behavior*, *51*(2), 301–307. https://doi.org/10.1111/sltb.12705 [67]	Mental Health First Aid (MHFA)	Mental health education; psychological first aid; psychological intervention skill training
Ryan, A. S., & Epstein, I. (1987). Mental health training for Southeast Asian refugee resettlement workers. International Social Work, 30(2), 185-198. https://doi.org/10.1177/002087288703000209 (Original work published 1987) [68]	Does not describe the name of the training/intervention	Mental health education; evidence-based intervention models; psychological intervention skills training; interventions for family caregivers; training aimed at reducing health disparities
Shields-Zeeman, L., Pathare, S., Walters, B. H., Kapadia-Kundu, N., & Joag, K. (2017). Promoting wellbeing and improving access to mental health care through community champions in rural India: the *Atmiyata* intervention approach. *International journal of mental health systems*, *11*, 6. https://doi.org/10.1186/s13033-016-0113-3 [45]	Atmiyata program	Instructional techniques, psychological intervention skill training, mental health education
Sibeko, G., Milligan, P.D., Roelofse, M. et al. Piloting a mental health training programme for community health workers in South Africa: an exploration of changes in knowledge, confidence and attitudes. *BMC Psychiatry* 18, 191 (2018). https://doi.org/10.1186/s12888-018-1772-1 [49]	Does not describe the name of the training/intervention	Mental health education; prevention; psychological intervention skill training
Smith, M. V., & Kruse-Austin, A. (2015). A gender-informed model to train community health workers in maternal mental health. *Evaluation and program planning*, *51*, 59–62. https://doi.org/10.1016/j.evalprogplan.2014.12.008 [13]	Does not describe the name of the training/intervention	Instructional techniques, mental health education, prevention, training aimed at reducing health disparities
Stacciarini, J. M., Rosa, A., Ortiz, M., Munari, D. B., Uicab, G., & Balam, M. (2012). Promotoras in mental health: a review of English, Spanish, and Portuguese literature. *Family & community* *health*, *35*(2), 92–102. https://doi.org/10.1097/FCH.0b013e3182464f65 [69]	Does not describe the name of the training/intervention	Mental health education
Tyagi, V., Khan, A., Siddiqui, S., Kakra Abhilashi, M., Dhurve, P., Tugnawat, D., Bhan, A., & Naslund, J. A. (2023). Development of a Digital Program for Training Community Health Workers in the Detection and Referral of Schizophrenia in Rural India. *The Psychiatric quarterly*, *94*(2), 141–163. https://doi.org/10.1007/s11126-023-10019-w [38]	The content of the digital training program was adapted from the COPSI (Community care for People with Schizophrenia in India) program manual	Instructional techniques, mental health education, prevention, training aimed at reducing stigma, training aimed at reducing health disparities
Weaver, A., & Lapidos, A. (2018). Mental Health Interventions with Community Health Workers in the United States: A Systematic Review. *Journal of health care for the poor and underserved*, *29*(1), 159–180. https://doi.org/10.1353/hpu.2018.0011 [70]	Does not describe the name of the training/intervention	Mental health education; evidence-based intervention models; prevention; psychological intervention skill training
Wennerstrom, A., Vannoy, S. D., 3rd, Allen, C. E., Meyers, D., O’Toole, E., Wells, K. B., & Springgate, B. F. (2011). Community-based participatory development of a community health worker mental health outreach role to extend collaborative care in post-Katrina New Orleans. *Ethnicity & disease*, *21* (3 Suppl 1), S1–51. [12]	The REACH NOLA Mental Health Infrastructure and Training Project (MHIT)	Instructional techniques, psychological intervention skills training, mental health education, prevention, training aimed at reducing health disparities
Wright, J., & Chiwandira, C. (2016). Building capacity for community mental health care in rural Malawi: Findings from a district-wide task-sharing intervention with village-based health workers. *The International journal of social psychiatry*, *62*(6), 589–596. https://doi.org/10.1177/0020764016657112 [71]	Mental Health in Zomba (MHiZ) Project	Mental health education, evidence-based intervention models, psychological first aid, prevention, psychological intervention skill training, interventions for family/caregivers, training aimed at reducing stigma, training aimed at reducing health disparities
